# Heme Oxygenase-1 as a Modulator of Intestinal Inflammation Development and Progression

**DOI:** 10.3389/fimmu.2018.01956

**Published:** 2018-09-12

**Authors:** Valentina P. Sebastián, Geraldyne A. Salazar, Irenice Coronado-Arrázola, Bárbara M. Schultz, Omar P. Vallejos, Loni Berkowitz, Manuel M. Álvarez-Lobos, Claudia A. Riedel, Alexis M. Kalergis, Susan M. Bueno

**Affiliations:** ^1^Millennium Institute on Immunology and Immunotherapy, Departamento de Genética Molecular y Microbiología, Facultad de Ciencias Biológicas, Pontificia Universidad Católica de Chile, Santiago, Chile; ^2^Departamento de Gastroenterología, Facultad de Medicina, Pontificia Universidad Católica de Chile, Santiago, Chile; ^3^Millennium Institute on Immunology and Immunotherapy, Facultad de Ciencias de la Vida, Departamento de Ciencias Biológicas, Universidad Andrés Bello, Santiago, Chile; ^4^Departamento de Endocrinología, Facultad de Medicina, Pontificia Universidad Católica de Chile, Santiago, Chile

**Keywords:** heme oxygenase-1, inflammatory bowel disease, carbon monoxide, infection, inflammation, nuclear factor erythroid 2-related factor 2, colorectal cancer, microbiota

## Abstract

Heme Oxygenase 1 (HMOX1) is an enzyme that catalyzes the reaction that degrades the heme group contained in several important proteins, such as hemoglobin, myoglobin, and cytochrome p450. The enzymatic reaction catalyzed by HMOX1 generates Fe^2+^, biliverdin and CO. It has been shown that HMOX1 activity and the by-product CO can downmodulate the damaging immune response in several models of intestinal inflammation as a result of pharmacological induction of HMOX1 expression and the administration of non-toxic amounts of CO. Inflammatory Bowel Diseases, which includes Crohn's Disease (CD) and Ulcerative Colitis (UC), are one of the most studied ailments associated to HMOX1 effects. However, microbiota imbalances and infections are also important factors influencing the occurrence of acute and chronic intestinal inflammation, where HMOX1 activity may play a major role. As part of this article we discuss the immune modulatory capacity of HMOX1 during IBD, as well during the infections and interactions with the microbiota that contribute to this inflammatory disease.

## Introduction

Heme oxygenase 1 (HMOX1) is an enzyme that catalyzes the first step of the oxidative degradation of the heme group, which is a rate-limiting reaction that releases the following molecules as by-products: carbon monoxide (CO), free iron, and biliverdin (which is lately reduced to bilirubin) (1). HMOX1 is composed of 288 amino acid residues with an active site located between the first two alpha-helixes (2). The exact reactions leading to the conversion of hemoglobin, hemin, and myoglobin into bilirubin were identified in 1969, when Tenhunen, Marver, and Schmid described that this reaction was catalyzed by a heme oxygenase enzyme, based on two observations. First, the reaction required a metal chelate and the formation of only one isomer of bile pigment, suggesting the participation of an enzyme in this reaction. Second, the insertion of two hydroxyl groups that indicated that the cleavage of the porphyrin ring was an oxidative reaction. Based on these observations, authors described that heme oxygenase stoichiometrically required NADPH and molecular oxygen to generate the same amount of carbon monoxide and bilirubin (3, 4). Few years later, Maines and Kappas described that, not only heme is a substrate for heme oxygenase, but also Cobalt protoporphyrin IX (CoPP). Using microsomal fractions, a NADPH-generating system and biliverdin reductase, the authors measured CoPP oxidation. These results suggested that CoPP can be bound to and oxidized by heme oxygenase, as well as is heme. On the contrary, although nonreactive metalloporphyrins, such as Ni-heme, bind to the enzyme these molecules are not oxidized by HMOX1 and inhibit its enzymatic activity (5). Another important metalloporphyrin that inhibits HMOX1 activity is tin-protoporphyrin (SnPP), a molecule that was early evaluated as a potential therapy for hyperbilirubinemia in newborns, due to its capacity of inhibit the formation of bile pigments (6). SnPP has the ability to strongly inhibit heme oxygenase activity by binding to the same site than does heme and CoPP, competing with these substrate molecules. At the same time, SnPP treatment increases the amount of heme oxygenase protein in rat liver cells, probably, as a compensatory response to the reduced activity of the enzyme (7).

## Role of HMOX1 under physiological conditions

Although HMOX1 is expressed in all mammalian tissues at basal levels, expression levels of this enzyme can significantly increase in cells or tissues where red blood cells or hemoglobin are degraded by macrophages, such as the spleen, liver, bone marrow, and kidney. In these tissues HMOX1 expression can be induced either by substrate (heme) or by other physical and chemical stimuli, including metalloporphyrins (8), oxidative stress, pathogen-associated molecular patterns (PAMPs), and cytokines. The enzyme localization was first described in the microsomal fraction of spleen and liver cells of Sprague-Dawley rats (3). The association with the endoplasmic reticulum (ER) membrane under physiological conditions was later confirmed for this enzyme (9). However, under stress conditions, HMOX1 is capable of translocating to other cellular membranous compartments. Under lipopolysaccharide (LPS) and heme treatments, HMOX1 is found associated with cytochrome c-containing fractions, suggesting a possible localization in mitochondria. Stimulation with LPS, heme and hypoxia resulted in localization in low-density fractions, with high expression of caveolin-1, the principal structural component of caveolae or lipid rafts. Furthermore, HMOX1 also colocalized with NADPH:cytochrome P-450 reductase and biliverdin reductase, suggesting a functional role of HMOX1 in lipid raft activity (10). HMOX 1 nuclear localization has also been described in astroglial cells during differentiation. Confocal microscopy showed that HMOX1 at day 7 is localized in cytosol and perinuclear region, moving to the nucleus and nucleolus after 2 and 3 weeks of differentiation (11). Furthermore, biliverdin reductase has been localized in nuclear fraction of rat kidney, in response to HMOX1 inducers, LPS and bromobenzene. These data suggest a dynamic localization for HMOX1 and that this molecule can display enzymatic activity at the various sites (12).

**Figure 1:**
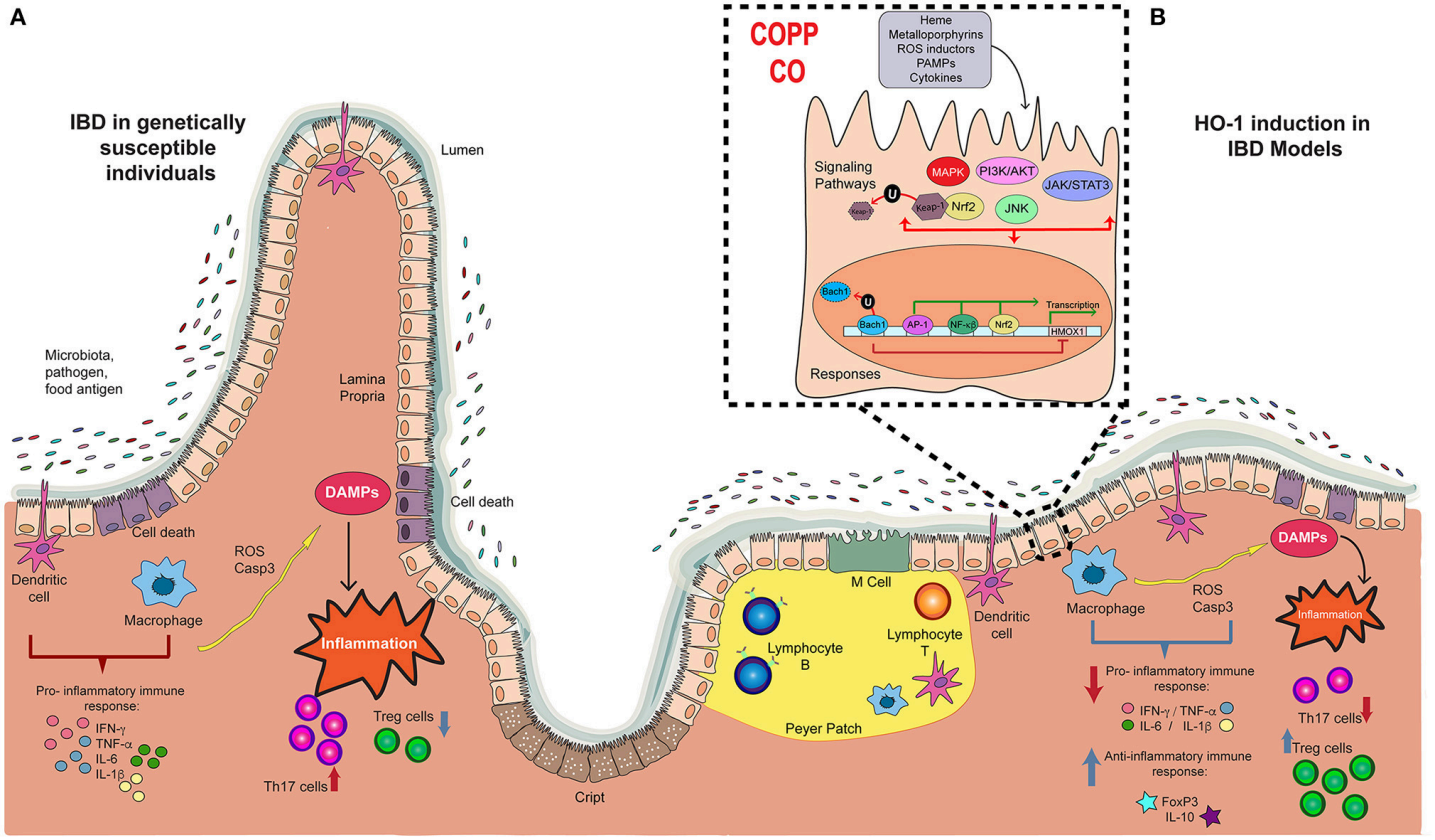
Players in the development and progression of IBD and the potential therapeutic effect of Hemoxigenase-1. **(A)** The pathology of IBD in genetically susceptible patients is characterized by the recognition of microbiota, pathogens and food antigens developing a pro-inflammatory immune response, with an augmented production of pro-inflammatory cytokines such as IFN-g, TNF- α, IL-6, and IL-1b. This response triggers tissue damage by the production of reactive oxygen species (ROS) and Caspase-3, which releases damage-associated molecular patterns (DAMPs) that induces the intestinal inflammation, with high Th17 profile T cell number, and reduced Treg cells. **(B)** In some IBD models, treatment with CoPP or CO reduces the pro-inflammatory immune response described above, and induces the production of anti-inflammatory molecules, such as FoxP3 and IL-10. Consequently, cell death is reduced, diminishing Th17 response. Also, CoPP or CO increase number of Treg cells. This anti-inflammatory effect result in reduced colitis, suggesting that HMOX1 activity is an important target in the development of new therapies for IBD.

Because of the wide range of molecules that can trigger the induction of HMOX1 expression, the complete network of gene regulation leading to HMOX1 expression is not yet fully understood. Nevertheless, there are some signaling pathways and transcription factors that have been well associated with the HMOX1 response (Figure 1B). The main regulators of *HMOX1* transcription described so far is the transcription factor Nrf2 [nuclear factor (erythroid-derived 2)-like 2] (13) and the inducible repressor Bach1 (BTB and CNC homology 1) (14). Indeed, the effect of CoPP over HMOX1 induction involves the participation of these two regulators (15). Nrf2 is a basic Leucine zipper protein that regulates the expression of antioxidant proteins, as the response to oxidative stress, including HMOX1 (16). Several are the stimuli and signaling pathways leading Nrf2 to induce HMOX1 expression. For instance, exposure to the flavonoid Orientin (Ori) can alleviate hydrogen peroxide-induced oxidative impairment in RAW264.7 cells, by induction of Nrf2/HMOX1 axis, through c-Jun N-terminal kinases (JNK) and phosphoinositide-3 kinase (PI3K)/protein kinase B (AKT) activation (17). Cytokines are another stimuli for HMOX1 expression (18) and it has been demonstrated that in human macrophages, IL-10 and IL-6-induced expression of HMOX1 required the activation of the signal transducer and activator of transcription 3 (STAT3) (19). On the contrary, Bach1 is a repressor of HMOX1 in physiological conditions, when Bach1 is absent, HMOX1 is constitutively expressed (14). Moreover, a deficiency of Bach1 protects against osteoarthritis and from oxidative stress-induced injury through the upregulation of HMOX1 (20, 21).

## Evidence supporting a role for HMOX1 in inflammation

The enzymatic activity of HMOX1 was initially associated with the immune response during inflammatory processes involved in organs rejection. Early on was observed that the mechanisms protecting xenografts from being rejected involved a rapid increase of HMOX1 expression by the endothelial and smooth muscle cells from mouse cardiac xenografts transplanted into rats (22). Two years later, experiments performed in a mouse-to-rat cardiac transplant model showed that the inhibition of HMOX1 activity by tin-protoporphyrin resulted in an earlier organ rejection, but when these same rats were additionally treated with exogenous CO, the long-term graft survival was restored (23). These studies demonstrated the important role that HMOX1 can play in the immune modulation involved in transplants, and that this immunomodulatory effect was driven by CO (23).

Tardif and collaborators demonstrated that Dendritic Cells (DCs) treated with lipopolysaccharide (LPS) and CO showed a diminished capacity to present antigens to T cells and this effect was due to a reduced fusion of endosomes and lysosomes, that is required for antigen presentation (24). Such reduced fusion of endosomes and lysosomes is probably caused by ATP reduction in the cell, as a result of impaired mitochondrial function caused by CO (25–27). The activation of adaptive immunity requires antigen presentation resulting in cytokine secretion, which finally derives in cell infiltration and inflammation. Therefore, impaired antigen presentation will result in reduced inflammation. Another of the HMOX1 effects is the induction of anti-inflammatory cytokines production, such as IL-10. However, the interplay between IL-10 and HMOX1 seems to be much more complex. For instance, in murine macrophages it has been shown that treatment with recombinant IL-10 induces, in a dose-dependent manner, HMOX1 production measured by Western Blot and that such an induction is mediated by the MAPK p38 activity (28), which is modulated by CO (29). In addition, HMOX1 induction has been reported to enhance macrophages polarization into an IL-10-producing anti-inflammatory (M2) phenotype (30).

The anti-inflammatory properties of HMOX1 have also conferred it with a protective capacity on different tissues that have suffered injury. For instance, hypoxia can induce the secretion of several pro-inflammatory cytokines in lungs, producing severe inflammation and structural changes of vessels. Consistently, transgenic mice that constitutively express HMOX1 in the lungs submitted to hypoxia showed that an increased HMOX1 expression resulted in less inflammation and reduced vessel hypertrophy, as compared with wild type mice (31). Furthermore, the pro-inflammatory cytokines and chemokines expressed in normal conditions were significantly suppressed in HMOX1 transgenic mice (31). Similarly, the anti-inflammatory effects of HMOX1 induction were observed in FcγRIIb^−/−^ mice, which are a model of systemic lupus erythematosus (SLE). These FcγRIIb^−/−^ mice spontaneously develop proteinuria and renal inflammation, which are delayed by the induction of HMOX1 or carbon monoxide treatment. Indeed, carbon monoxide administration decreased the expansion of CD11b^+^ cells, prevented the decline of CD4^+^ FoxP3^+^ regulatory T (Treg) cells and reduced the titers of anti-histone antibodies, as compared to untreated mice (32). It has been also reported that patients with SLE show reduced expression of HMOX1 in circulating monocytes, suggesting that in myeloid cells, the expression of HMOX1 could contribute to modulate the host inflammatory response (33). Moreover, several studies support the notion that HMOX1 overexpression in the brain and spinal cord of multiple sclerosis patients could play an important role in multiple sclerosis patients, which was also observed in the experimental autoimmune encephalomyelitis (EAE) model (34). It has been observed that multiple sclerosis patients show a decreased expression of HMOX1 in mononuclear cells from the peripheral blood during disease exacerbation, similar to SLE patients (33, 35). A recent study has suggested an association between HMOX1 and HMOX2 polymorphisms and the risk of developing multiple sclerosis in Spanish Caucasian men (36). Additionally, possible associations between variants in *HMOX-1* genes and the risk of Parkinson's disease have been also reported (37, 38). Furthermore, it has been also described that HMOX1 is up-regulated during essential tremor and restless legs syndrome, suggesting a possible link between these ailments and the activity of HMOX1 (39).

Several studies have also revealed the relationship between HMOX1 and the pathophysiology of both acute and chronic intestinal inflammation (40). In the following chapter, we will discuss the studies performed up to date that support this notion.

## Features of inflammatory bowel diseases

Chronic intestinal inflammation can be triggered by several conditions, such as infections and autoimmune diseases. It is characterized by immune cell infiltration, edema and loss of the epithelium structure (41). The most studied chronic intestinal inflammations are denominated Inflammatory Bowel Diseases (IBD). These are chronic inflammatory pathologies of unknown etiology, which includes Crohn's Disease (CD) and Ulcerative Colitis (UC). IBD usually begins in early adulthood, and the symptoms include recurrent diarrhea, abdominal pain and the presence of blood in the stool (42). As part of Crohn's disease, inflammation can occur in any section of the gastrointestinal tract, whereas ulcerative colitis is typically confined to the colon (42). Although in recent years there has been a significant improvement in understanding the pathophysiology of these pathologies, their incidence has significantly increased worldwide and their etiology remains unclear (41). Unfortunately, there is still no cure for IBD, and the benefits of surgery are usually only temporary (43). Currently, the treatment of IBD is based on drugs to reduce symptoms and inflammation, such as 5-aminosalicylic acid, corticosteroids, immunomodulators, and biological therapy (44). However, these treatments are only palliative and most of them can have significant side effects. For this reason, there is a broad interest to understand in more detail the pathophysiology of these diseases in order to identify new therapeutic targets.

Even though the specific cause of IBD is not clear, it is known that infections, genetic predisposition, and environmental factors can contribute to disease progression (45–52). Importantly, several studies on this field suggest that IBD is caused by an inappropriate immune response to enteric commensal bacteria in genetically susceptible hosts (53–56). Such an unbalanced immune response leads to a persistent and relapsing inflammation of different portions of the gastrointestinal tract (41). Supporting this hypothesis are genome-wide association studies that have identified approximately 200 inflammatory bowel disease-associated loci (57). Most of the genes that have been linked to IBD are related to host defense against infection, particularly to the interaction between the host mucosal immune system and microbes at the epithelial cell surface and inside the gut lumen (58). The best-known example is the association of CD with *nod2* variants (59). *nod2* encodes the primary receptor for muramyl dipeptide (MDP) and is essential for bacterial recognition. Defective *nod2* prevents the clearance of intracellular bacteria, due to a deficient antimicrobial response, which favors the activation of the immune response against the microbiota (60).

Dysregulation of immune response in the pathogenesis of IBD is also associated with an imbalance between pro- and anti-inflammatory molecules involved in both innate and adaptive immunity, leading to the development of excessive and chronic inflammation (61). In physiological conditions, the intestinal mucosa represents a mechanical barrier between immune system and microorganisms (62) and the abundant immunogenic stimuli induce infiltration of lymphocytes, monocytes, mast cells, and eosinophils (63). This physiological response is a tightly regulated phenomenon, which is important to keep the integrity of the gastrointestinal barrier (62). However, under certain pathological conditions this mechanism of control fails, leading to a persistent detrimental inflammation of the digestive tract. Along these lines, it has been reported that patients with IBD display an activated mucosal immune system, with high infiltration of plasma cells, neutrophils and macrophages, accompanied by overexpression of pro-inflammatory cytokines and various chemokines that promote the recruitment of inflammatory cells (64). Together, cytokines, chemokines, reactive oxygen species and other mediators result in exacerbated local inflammation and tissue damage (42) (Figure 1A).

Chronic inflammatory response in CD is characterized by an abnormal Th1 and Th17 immune responses, which are triggered by increased mucosal levels of cytokines, including tumor necrosis factor-alpha (TNF-α), interferon-gamma (IFN-γ), IL-12, IL-17, and IL-18 (Figure 1A). In contrast, the production of IL-4, IL-5, and IL-13 is increased in UC, which leads to a Th2-polarized immune response. Although in both disorders the immune response is different, genetically susceptible individuals have an inappropriate mucosal immune response against their gut microbiota, mediated by an excessive activation of effector T cell subsets and/or a deficiency of Treg cells (65) (Figure 1A). Indeed, Treg cells play a crucial role in the maintenance of intestinal homeostasis through TGF-β- and IL-10-dependent mechanisms. For instance, IL-10 is a potent anti-inflammatory cytokine produced by both Treg and non-Treg cells, capable of suppressing the production of pro-inflammatory mediators to limit the inflammatory response. In fact, IL-10-deficient mice are highly susceptible to colitis, due to aberrant immune response to commensal bacteria. This abnormal response is more severe when combined with a deficiency of TGF-β signaling (66). Previous studies have indicated that IL-10 production is suppressed in the intestine during IBD. However, the molecular and cellular mechanisms behind the reduction of IL-10 secretion observed during IBD have not yet been fully understood. In addition, it has been suggested that IL-10 could induce other molecules with anti-inflammatory activity by a positive feedback circuit, which leads to the amplification of this response and the inhibition of intestinal inflammation. Abnormal low levels of IL-10 during these diseases could account for the inflammatory imbalance in the intestinal tissue (67).

As described above, several studies have associated IL-10 production and HMOX1 activity with the development of IBD (67). Therefore, it has been important to determine the contribution of the HMOX1 enzyme to the advancement of intestinal inflammation. Under healthy conditions, HMOX1 expresses in the intestine at low levels, but it is significantly induced upon triggering of an inflammatory state (68). HMOX1 mRNA expression and HMOX1 protein production are increased in inflamed colonic mucosa of UC patients. In addition, HMOX1 expression is also decreased in mononuclear cells from colonic submucosa from UC patients, as compared to normal mucosa from healthy subjects (69). Similar data were obtained during the occurrence of three different inflammatory conditions: *Helicobacter pylori*-positive gastritis patients, active UC and active CD. Immunohistochemical staining showed a higher expression of HMOX1 in gastric mucosa of *Helicobacter pylori*-positive gastritis patients and in colonic mucosa of active UC and active CD patients, as compared to healthy controls (70). In the next section, studies performed to evaluate the effect of HMOX1 modulation in IBD will be discussed.

## Potential therapeutic use of HMOX1 modulation and CO production for IBD

The contribution of HMOX1 to the prevention or reduction of intestinal inflammation has been shown in different models of IBD, which are summarized in Table 1. It has been reported that in the model of colitis induction by the administration of Dextran Sulfate Sodium (DSS) an increase in the expression of HMOX1 led to an amelioration of the intestinal inflammation. Induction of HMOX1 by intraperitoneal injection of CoPP at day 1 and 3 after DSS treatment significantly reduced the intestinal histological damage as compared to control animals (72). Another study also described the cytoprotective role of HMOX1 in the model of colitis induced by DSS in mice. In this study, a treatment with hydrogen-rich water was performed in mice treated with DSS, resulting in decreased inflammation and disease score, increased level of anti-oxidative markers and reduced production of inflammatory molecules. Importantly, it was shown that this treatment up-regulated HMOX1 expression (78). In support of this observation, another study showed that mice treated with the HMOX1 inducer hemin previous to the induction of DSS colitis displayed reduced histological damage, which was associated to an increased expression of HMOX1. Furthermore, a decreased proportion of Th17 cells and increased number of Treg cells were found in mesenteric lymph nodes (MLN) and spleen of hemin-pretreated mice that were exposed to DSS (77).

**Table 1:** Protective effect of HMOX1/CO in inflammatory intestinal models.

**Model/disease**	**Treatment**	**Route of administration**	**Effect**	**Animal model**	**Reference**
TNBS-induced colitis	SnMP (inhibitor of HMOX1 activity)	Subcutaneous	Increase of colonic damage Enhance ROS and iNOS	Rat	(71)
DSS-induced colitis	CoPP (inductor of HMOX1 expression	Intraperitoneal	Reduction of IFNγ in mLN Reduction of apoptotic epithelial cells in colon	Mouse	(72)
IL-10^−/−^ mice	CO gas	Air exposure	Reduction of IL-12 p40 and TNF intestinal secretion	Mouse	(73)
Necrotizing enterocolitis	CO gas	Air exposure	Reduction of serum IL-1β, TNFα, and nitrites	Rat	(74)
TNBS-induced colitis	CO gas	Intrarectal	Reduction of ulcer area and wet colon weight Inhibition of MPO activity, TBA-reactive substances, and CINC-1 expression	Rat	(75)
TNBS-induced colitis	CORM-3 (CO releasing molecule 3)	Intraperitoneal	Reduction of MPO activity, TNFα, IFNγ, and IL-17A expression	Mouse	(76)
DSS-induced colitis	Hemin (inductor of HMOX1 expression	Intraperitoneal	Decrease of colitis symptoms and histological damage Decrease in proportion of Th17 cells and increased number of Treg in spleen and mLN	Mouse	(77)

*ROS, reactive oxygen species; mLN, mesenteric lymph nodes; MPO, myeloperoxidase; TBA, thiobarbituric acid; CINC-1, cytokine-induced neutrophil chemoattractant 1*.

Another mouse model of colitis induction, in which the effect of HMOX-1 was evaluated, is the treatment with 2,4,6-trinitrobenzene sulfonic acid (TNBS). Both HMOX1 mRNA expression and enzymatic activity were induced after TNBS treatment. Further, when the HMOX1 inhibitor tin mesoporphyrin (SnMP) was used in these assays, HMOX1 activity was reduced and colonic damage increased (71).

An analysis of the role of HMOX1 and IBD has been also performed using patient samples, including both *in vitro* and *in vivo* experiments. In these studies, IBD patients showed a heterogeneous distribution of HMOX1 expression in the colonic mucosa, as compared to patients with intestinal damage produced by conditions different from IBD, such as diverticulitis. During these ailments the expression of HMOX1 was shown to be highly increased in epithelial cells of colonic crypts and in macrophages (72). Consistently, some drugs used in patients with IBD have shown to have a beneficial effect through the induction of HMOX1. For example, it was demonstrated that 5-aminosalicylic acid (5-ASA), an anti-inflammatory drug often used to treat IBD, reduces the TNBS-provoked colonic inflammatory injury in rats, by increasing colonic HMOX1 activity (79). Another example is Tranilast, a mastocyte stabilizer used in some countries as IBD treatment. The simultaneous intrarectal administration of DSS and Tranilast to C57BL/6 mice resulted in an attenuated colitis, produced by low pro-inflammatory cytokines, increased anti-inflammatory cytokines and promoted HMOX1 expression (80). In another field, *in vitro* studies have demonstrated that treatment with plant extracts of *Atractylodes macrocephala* (AM) or *Taraxacum herba* (TH) increased HMOX1 production. Oral pre-administration of AM and TH can rescue mice from DSS-induced colitis by inhibiting inflammatory mediators via inactivated extracellular signal regulated kinase (ERKs) and repressed NFκB and signal transducer and activator of transcription 3 (81).

Given that HMOX1 might be involved in the immune processes leading to intestinal inflammation, CO has been considered as a therapeutic tool to reduce the symptoms of this disease (82). The first evidence supporting that CO could work as a therapy for IBD is the observation that smoking can work as a protective factor in UC patients (83, 84). It was shown that exposure to CO ameliorates chronic intestinal inflammation in IL-10^−/−^ mice by interfering with the IFN-γ signaling that mediates inflammation in this model (73). Similar results were found in another murine model of IBD. In this case, mice were treated with TNBS to induce intestinal inflammation, and CO gas was administrated intrarectally at 200 ppm per day. TNBS- and CO-treated mice showed significantly smaller ulcers in colon, less colon weight and reduced myeloperoxidase (MPO) activity. Further, CO-treated mice showed reduced thiobarbituric acid (TBA)-reactive substances and cytokine-induced neutrophil chemoattractant 1 (CINC-1) expression in colonic mucosa, as compared with TNBS-air treated mice (75). In a necrotizing enterocolitis rat model, inhaled CO gas also protected against intestinal inflammation and decreased levels of TNF-α (74). However, exposition to CO is not the only way to administrate low doses of CO. Carbon monoxide-releasing molecules (CORM) have been widely used to corroborate the effectiveness of CO. A study showed that intraperitoneal CORM-3 injection in mice for 3 days prior to TNBS treatment attenuated distal colon damage and histological score. In an *in vitro* approximation, CORM-3 treatment reduced IFN-γ and TNF-α in CD4^+^ T cells stimulated with anti-CD3/CD28 antibodies (76).

The evidences listed in Tables 1, 2 support the important role of HMOX1 during the pathology of IBD in animal models and in humans. However, to elucidate the molecular mechanism of HMOX1 activity would help to understand those effects and to the development of new potential therapies for IBD (Figure 1B).

**Table 2:** Protective effect of different compounds in inflammatory intestinal models that involves HMOX1 pathways.

**Model/disease**	**Treatment**	**Route of administration**	**Effect**	**Animal model**	**Reference**
TNBS-induced colitis	5-ASA (5-aminosalicylate)	Intracolonic	Reduction of macroscopic colonic inflammation, MPO activity, and TNFα levels	Rat	(79)
DSS-induced colitis	Tranilast	Intrarectal	Amelioration of clinical symptoms of colitis Decrease in number and degranulation of mast cells in colon Decrease of TNFα, IFNγ, and IL-6 expression in colon Increase of IL-10 expression in colon	Mouse	(80)
DSS-induced colitis	HRW (Hydrogen-rich water)	Intraperitoneal	Attenuation of macroscopic and microscopic colonic damage scores Reduction of MDA and MPO, expression in colon. Reduction of TNFα, IL-6, and IL-1β in blood	Mouse	(78)
DSS-induced colitis	*Atractylodes macrocephala* and *Taraxacum herba* plant extracts	Gavage	Inhibition of iNOS, IL-1β, and TNFα expression in colon	Mouse	(81)
DSS-induced colitis	Hyperoside (flavonoid)	Gavage	Reduction in TNFα, IL-6, COX-2 and NF-κB p65 expression in colon	Mouse	(85)
DSS-induced colitis	Ginger active compound (6-shogaol)	Oral	Reduction of TNFα, IL-6, IL-1β, and iNOS expression in colon	Mouse	(86)
Adoptive T-cell transfer model of chronic colitis	Quercetin (flavonoid)	Oral	Reduction of TNFα, IL-23, IL-12p40 expression in lamina propria lymphocytes	Mouse	(87)
Acetic acid-induced colitis	*Ziziphus spina-christi* fruit extract	Gavage	Reduction of LPO, NO and MPO activity Induction of antioxidant activity (SOD, CAT, GPx, GRd)	Rat	(88)
DSS-induced colitis	Alpinetin (flavonoid)	Oral	Reduction of macroscopic damage scores Upregulation of occludin, zonula occludens-1 and SOD expression Downregulation of claudin-2	Mouse	(89)

*MDA, Malonaldehyde; COX-2, cyclooxygenase-2; LPO, lipid peroxidation; SOD, superoxide dismutase; CAT, catalase; GPx, glutathione peroxidase; GRd, glutathione reductase*.

## Interplay between HMOX1 and microorganisms in the gastrointestinal tract

An important cause of intestinal inflammation is the infection by pathogenic bacteria, such as *Salmonella enterica*. Although little is known about the exact effect of HMOX1 on bacterial infections, it has been described that HMOX1 plays a role in the clearance of *Salmonella* by the immune system. For instance, in an acute inflammation model, where C57BL/6 mice were treated with streptomycin to eliminate the microbiota, prior to *Salmonella enterica* serovar Typhimurium (*S*. Typhimurium) oral infection, treatment with CoPP reduced the presence of *Salmonella* DNA in the mesenteric lymph nodes, lamina propria, liver and spleen (90). Furthermore, *in vitro* depletion of HMOX1 in murine macrophages reduced these cells bactericidal activity against *E. coli, Enterococcus faecalis* and *S. Typhimurium*. These data suggest that the observed CO effect is due to the capacity to promote bacterial clearance in phagocytic cells associated to the intestine (90). A recent *in vitro* study supports these findings, in which the bacterial clearance capacity of murine and human macrophages was tested. Specifically, it was observed that in HMOX1-deficient mice, macrophages have bacterial killing defects that were restored with the administration of CO. Further, CO treatment results in an enhanced bacterial clearance capacity in mouse and human macrophages (91). In contrast, CO has no effect on bactericidal activity of macrophages isolated from NALP3-deficient and caspase3-deficient mice. Consequently, the capacity of macrophages of killing bacteria depends on CO-mediated inflammasome activation (91). However, apparently contradictory results were observed in murine macrophages transfected with *hmox1* shRNA. In these macrophages, intracellular *S*. Typhimurium survival was reduced upon *hmox1* knockdown, an effect attributed to iron availability inside the cell (92).

Other bacteria that can be affected by the action of HMOX1 in the intestine are members of the intestinal microbiota. Recent research has revealed that alterations in the intestinal microbiota play a key role in the development of colitis and other inflammatory conditions of the gut (55). For example, there are studies that support an interaction between the host and molecules produced by commensal bacteria. Some of these are Short Chain Fatty Acid (SCFA), such as acetate, butyrate and propionate, which can promote an anti-inflammatory response in the intestine (93).

In addition, when C57BL/6 mice are exposed to specific pathogen free (SPF) enteric microbiota after been raised under germ free conditions, colonic HMOX1 expression is increased almost 6 times, and this induction depends on IL-10 and Nrf2 (90). These data support the notion that HMOX1 not only works by inducing an anti-inflammatory cytokine profile, but also by interacting with intestinal microbiota.

As well as mammals, other organisms have enzymes with functions equivalent to HMOX1. It has been described that some bacteria express heme-degrading proteins that act as a complex group of molecules that work together to catalyze heme degradation (94, 95). Some enteric pathogens and commensal bacteria express this HMOX1 homolog, which may affect intestinal immune response. *Corynebacterium diphteriae* has a heme-oxygenase (HmuO) that has 33% sequence identity and 70% of homology with human HMOX1 (96). Most of the known bacterial HO are described for pathogens, such as *Yersinia pestis* (Hmu), *Neisseriae spp*. (HemO)*, Pseudomonas aeruginosa* (PigA), *Mycobacterium tuberculosis* (MhuD), and *Staphylococcus aureus* (IsdG, IsdI). All of these bacteria harbor genes for HO that encode functional proteins (94, 97–101).

**Figure 2:**
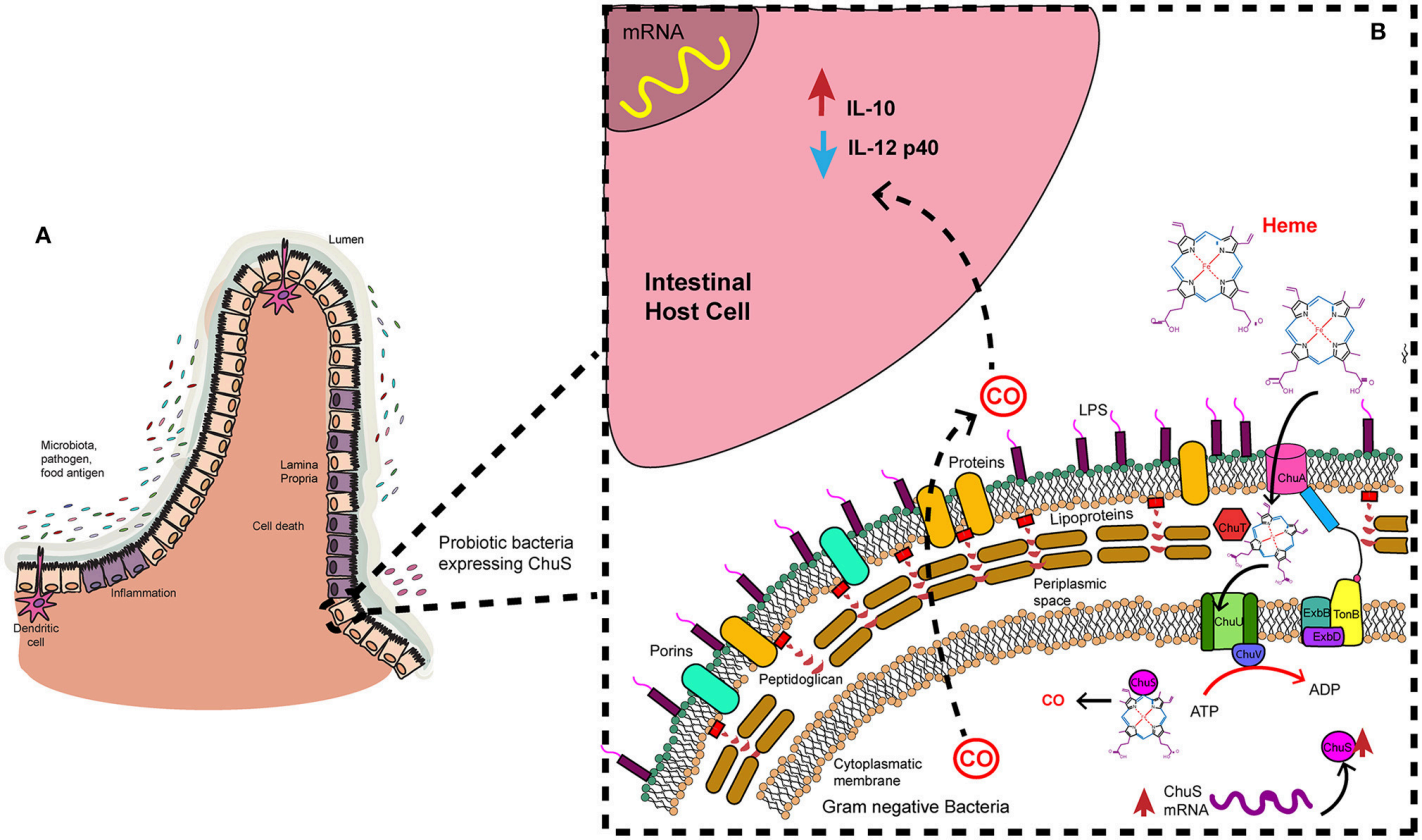
Heme uptake and degradation by gram negative bacteria. Microbiota is in constant interaction with the epithelial cells. **(A)** In an inflammation context in the intestine some bacteria may modulate immune response. **(B)** That is the case of a probiotic strain with ChuS. These bacteria have an outer membrane receptor (ChuA) catches specifically heme group. Periplasmic protein (ChuT) binds substrate and carry it to inner membrane protein (ChuU), where ATPase direct the transport of the substrate to the cytoplasm. There, HMOX1 analog (ChuS) binds heme group and catalyzes its degradation. CO is a product of this reaction and may be released to the environment where it exerts its anti-inflammatory effect on host cells.

Some strains of *E. coli* have a heme uptake gene cluster that is required for harnessing available iron in the environment, presented as heme group. Bacterial analog of HMOX1 called ChuS is encoded by *chu*S. Metabolic products are the same as human HMOX1. For instance, HO enzyme of *E. coli* O157:H7 produces CO efficiently, despite having a different structure from HMOX1. Additionally, enteric bacteria as *Enterobacter* and *Shigella* may have similar proteins (95, 102). Other proteins that are involved in heme uptake machinery are heme receptor called ChuA (103) present in the outer membrane, periplasmic protein ChuT which binds to heme, inner membrane protein ChuU and the ATPase ChuV (104) (Figure 2B).

A recent study described the anti-inflammatory action of an *E. coli* strain isolated from mice gut, named NC101, which has *chuS* gene. In mono-association studies, germ-free SvEV/129 IL-10 deficient mice were treated with this bacterial strain. A correlation was found between *chuS* expression on feces and the progression of inflammation. In addition, composition of intestinal bacteria was altered when C57BL/6 wild type mice were exposed to carbon monoxide for 14 days. Also, *chuS* expression was increased in that point. Interestingly these changes returned to baseline after 14 days without CO exposure. Additionally, when BMDMs were cultured with an *E. coli* NC101 strain that overexpresses *chuS* pro-inflammatory cytokine IL-12p40 production was decreased. On the other hand, IL-10 production increased, promoting an anti-inflammatory scenario. These changes in cytokines expression were promoted by a soluble factor released by bacteria. Finally, CO abundance found in liver of mice infected with the strain that overexpresses *chuS* was higher than in those infected with a strain that lacks *chuS*. Moreover, in the first group of mice mentioned before expression of HMOX1 and *Nqo1* were significantly increased same as IL-10 production (105).

These *in vitro* and *in vivo* findings contribute to the hypothesis that ChuS may be used to modulate immune response in an inflammation context (Figure 2A). A probiotic strain with ChuS enzyme may be able to decrease inflammation in the gut by secreting CO as a product of heme uptake process. CO would act in host cells (gut epithelium) modulating IL-12p40 and IL-10 expression and production. Consequently, inflammation will be ameliorated, leading to an anti-inflammatory scenario (Figure 2B).

## HMOX1 as a cancer target

Another aspect of intestinal inflammation where HMOX1 is involved is cancer. HMOX1 has been associated with different types of cancer (106), including prostate cancer (107), bladder cancer (108), skin cancer (109), and colorectal cancer (CRC). The latter is an important issue due to all the evidences that support the hypothesis that HMOX1 is a key immune modulator in intestine. It has been demonstrated that HMOX1 is highly expressed in CRC (110) and high expression of HMOX1 in small-intestine adenocarcinomas is associated with lower pT (primary tumor) category, less pancreatic invasion and longer overall survival than those with low HMOX1 expression (111). Another study also showed that HMOX1 was over-expressed in human invasive CRC, associated with longer overall survival time. Using 1,2-dimethylhydrazine (DMH)-induced CRC animal model, the authors showed that HMOX1 expression is enhanced as tumor progress, and that this effect is due to the induction of cell cycle arrest and apoptosis (112). *In vitro* studies also showed augmented expression of HMOX1 in primary colon cancer tissues and HCT116 colon cancer cells compared with normal surrounding tissue and with normal epithelial cell line, respectively. Moreover, treatment with CORM-3 inhibited cell proliferation of HCT116 cells (113). An important feature of intestinal immune response are macrophages. Particularly, CX3CR1^+^ macrophages contribute to intestinal homeostasis, controlling T cell response to commensal bacteria (114, 115). CX3CR1 deficient mice could not resolve intestinal inflammation in azoxymethane (AOM)/DSS-induced colitis-associated cancer and have higher colitis score and polyps, compared with wildtype mice. These authors showed that CX3CR1 deficient mice has lower HMOX1 expression in adenomatous colon tissues, and treatment with CoPP restored the recovery reducing intestinal inflammation and preventing carcinogenesis (116).

Based on all these evidence, HMOX1 has been proposed as a target for cancer treatment (106), including its inducible repressor BACH1 (117), or its positive regulator Nrf2 (118).

## Concluding remarks

Since HMOX1 was originally described, strong evidence has supported the notion that this enzyme is a major modulator of immune response in several pathologies. Given that the number of IBD cases is increasing worldwide, it is very important to bring to light the etiology of these chronic diseases and new therapeutic alternatives. The evidences discussed in this review support the notion that HMOX1 activation reduces inflammation in the gut in several animal models of IBD, through the induction of anti-inflammatory cytokines pathways. Importantly, it has been demonstrated an association between IBD development and genes involved in the proper immune response to infecting microbes. Although the exact relation between HMOX1 and microorganisms colonizing—or infecting—the gastrointestinal tract is not totally elucidated yet, it is interesting that enzymes with similar functions than HMOX1 have been described in bacteria, particularly ChuS. Importantly, this bacterial enzyme has been associated with the induction of an anti-inflammatory environment in a mouse colitis model (105). Therefore, intestinal bacterial might be contributing to prevent intestinal inflammation through the byproducts of HMOX1 enzymatic activity. Furthermore, intestinal infections are an important factor in the development of IBD (51) and most of the evidence available to date supports the notion that HMOX1 promotes bacterial clearance, diminishing intestinal inflammation (90). Moreover, it has been demonstrated that CO can interact directly with heme groups of the bacterial electron transport chain, suggesting that HMOX1 has an effect in bacterial infections not only through the modulation of the immune response, but by the direct interaction with the pathogen (119). In another field, HMOX1 has also been associated with colorectal cancer, being suggested as a novel target for cancer therapy (106).

Because of the existing evidence of HMOX1 role in intestinal inflammation, many therapy targets can be proposed. Given that most of the anti-inflammatory and cytoprotective effects of HMOX1 have been attributed to CO, this could be an effective treatment. However, it is well known that CO in mammals is toxic from determined concentrations. For these reasons, it is very important to elucidate the exact mechanisms by which HMOX1 and CO are exerting all the effects described here, in order to design novel therapeutic strategies to deliver CO in a safe manner.

## Author contributions

All authors listed have made a substantial, direct and intellectual contribution to the work, and approved it for publication.

### Conflict of interest statement

The authors declare that the research was conducted in the absence of any commercial or financial relationships that could be construed as a potential conflict of interest.
